# Exploring invisibility and epistemic injustice in Long Covid—A citizen science qualitative analysis of patient stories from an online Covid community

**DOI:** 10.1111/hex.13518

**Published:** 2022-05-12

**Authors:** Jane Ireson, Amy Taylor, Ed Richardson, Beatrice Greenfield, Georgina Jones

**Affiliations:** ^1^ Centre for Psyhcological Research Leeds Beckett University Leeds UK; ^2^ Weston Park Cancer Centre Sheffield Teaching Hospitals NHS Foundation Trust Sheffield UK; ^3^ Department of Medical Imaging University of Exeter Exeter UK

**Keywords:** epistemic injustice, invisibility, lived experience, Long Covid, person‐centred care, qualitative, United Kingdom

## Abstract

**Background:**

In 2020, the long‐lasting effects of the Covid‐19 virus were not included in public messages of risks to public health. Long Covid emerged as a novel and enigmatic illness with a serious and life‐changing impact. Long Covid is poorly explained by objective medical tests, leading to widespread disbelief and stigma associated with the condition. The aim of this organic research is to explore the physical and epistemic challenges of living with Long Covid.

**Methods:**

Unlike any previous pandemic in history, online Covid communities and ‘citizen science’ have played a leading role in advancing our understanding of Long Covid. As patient‐led research of this grassroots Covid community, a team approach to thematic analysis was undertaken of 66 patient stories submitted online to covid19‐recovery.org at the beginning of the Covid‐19 pandemic between April and September 2020.

**Results:**

The overriding theme of the analysis highlights the complexities and challenges of living with Long Covid. Our distinct themes were identified: the life‐changing impact of the condition, the importance of validation and how, for many, seeking alternatives was felt to be their only option.

**Conclusions:**

Long Covid does not easily fit into the dominant evidence‐based practice and the biomedical model of health, which rely on objective indicators of the disease process. Patient testimonies are vital to understanding and treating Long Covid, yet patients are frequently disbelieved, and their testimonies are not taken seriously leading to stigma and epistemic injustice, which introduces a lack of trust into the therapeutic relationship.

**Patient Contribution:**

The research was undertaken in partnership with our consumer representative(s) and all findings and subsequent recommendations have been coproduced.

## 1 BACKGROUND

As of March 2022, there have been more than 483 million cases of Covid‐19, and over 6 million deaths worldwide.^1^ The frightening speed at which Covid‐19 took hold in 2019/2020 called for immediate action to minimize infections and deaths, yet an acute disaster response, as seen in the United Kingdom (UK), inadvertently created ‘Covid's paradox’.^2^ Despite there being numerous examples of the devastating long‐lasting effects of other viruses, including Covid‐19's predecessor severe acute respiratory syndrome,^3^ the notion of Long Covid was initially entirely dismissed and not counted.^4^, ^5^ Changes introduced to the UK's healthcare systems to respond to Covid‐19 paradoxically made the system less fit for the purpose of managing what has been called a ‘mass disabling event’ in the emergence of Long Covid.^6^ Long Covid is a patient made term used to describe symptoms that persist beyond the initial illness.^7^ The National Institute for Health and Care Excellence (NICE) defines Long Covid as the symptoms that continue or develop after acute Covid‐19 infection and which cannot be explained by an alternative diagnosis.^8^

The UK's Office for National Statistics shows that the estimated number of people with self‐reported Long Covid is steadily rising and currently stands at 1.5 million living with the condition in the UK in January 2022.^9^ The symptoms of Long Covid commonly include fatigue, breathlessness, chest pain, postexertional malaise, autonomic nervous system disruption and cognitive dysfunction among others and can cause episodic, hidden disability as Long Covid symptoms are poorly explained by objective medical tests.^10^ Within an era of evidence‐based practice Long Covid has been doubly challenging, for healthcare professionals (HCPs) working in a strained system who feel disempowered to provide answers, and for patients who feel invisible and are vulnerable to stigma and epistemic injustice.^11^ Epistemic injustice occurs when patients experience an unjustified discrediting as unreliable informants of their own illness experiences.^12^

This study is unique, in that it is the first citizen science qualitative study in the UK where all the data has originated from, and the analysis is driven by members of an online grassroots Covid‐19 community. Citizen science—the active and voluntary participation of the public in research has been invaluable in the pandemic, with studies such as the smartphone Zoe COVID Symptom Study app.^13^ The Covid Recovery (https://covid19-recovery.org/) website went live in April 2020 and was the first public‐facing website in the UK where patients could share their own unprompted stories of Covid‐19. As Long Covid was characterized online,^5^ this study gives a unique insight into the lived experience of Long Covid through the lens of an online grassroots Covid‐19 community, without any recruitment to a formal research study. The aim of this study was to provide a coproduced analysis of patient stories submitted to the Covid Recovery Collective,^5^ to further explore not only the physical nature of the condition but the epistemic challenges of the lived experience of Long Covid.

## 2 MATERIALS AND METHODS

A qualitative approach underpinned by phenomenology was adopted to enable the researchers to describe and understand the participants' health‐illness experiences during the early stages of the Covid‐19 pandemic.^14^ Site ownership permitted access to the stories submitted to the website and approval for the research was granted by the Leeds Beckett University ethics committee. Individuals submitted their stories by an online form, requesting: ‘In your own words, tell us your own experience of Covid‐19 and your ongoing recovery. Please try and include any symptoms and a sense of a timeline and the start date of your illness’.

**Figure A1:**
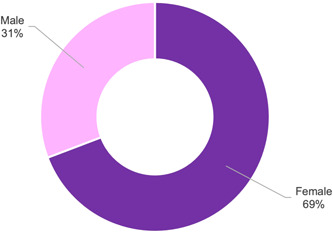
Percentage of submissions by gender.

**Figure A2:**
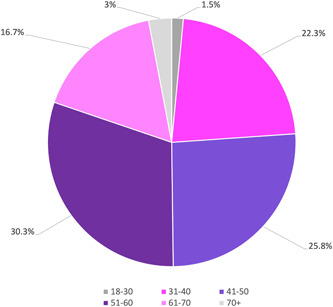
Percentage of submissions by age.

**Figure A3:**
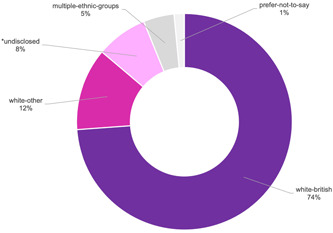
Percentage of submissions by ethnicity.

**Figure A4:**
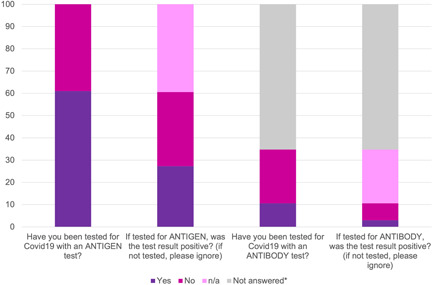
Percentage of submissions that had either a Covid‐19 ANTIGEN or ANTIBODY test and results.

Those stories with research consent submitted between April and November 2020 were included in the analysis, resulting in 66 individual stories with a mix of age, gender, ethnicity and Covid‐19 status (see Figures A1, A2, A3, A4). The time frame selected marked the beginning of the site's inception, through to 30 November 2020 after submissions had surpassed 60 with the perception this number would provide a rich narrative. This period captured the start of the pandemic, through to the middle of the UK's second national lockdown.

**Figure 1:**
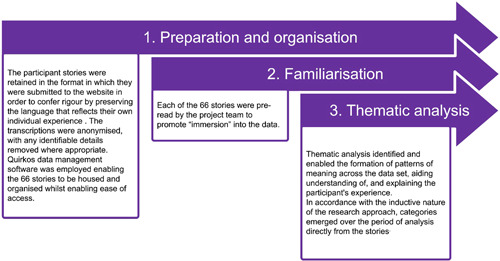
Three‐step process of data analysis.

The project team collectively recognized the value of involving individuals with different professional and Covid‐19 backgrounds in the process of coanalysis and coproduction to generate findings that provided ‘real life’ meaning.^15^ Thematic analysis using the methods outlined by Braun and Clarke^16^ was undertaken by four project team members (B. G., J. I., E. R., A. T.), coalesced with full project team meetings with G. J. (Table 1). A three‐stage process of analysis was employed by the project team to ensure the method taken by each member was structured, methodical, ethical and rigorous^17^, ^18^, ^19^, ^20^, ^21^, ^22^ (Figure 1).

**Table 1:** Project team expertize/background

Team member	Expertize/background
B. G.	Consumer representative (Covid‐19 diagnosis & long‐standing myalgic encephalomyelitis)
E. R.	Consumer representative (Covid‐19 diagnosis), website cocreator/owner, researcher
G. J.	Health psychologist, mixed‐method researcher
J. I.	Consumer representative (Covid‐19 diagnosis), website cocreator/owner, specialist nurse, researcher
A. T.	Therapeutic radiographer, qualitative researcher, coproduction specialist

**Figure 2:**
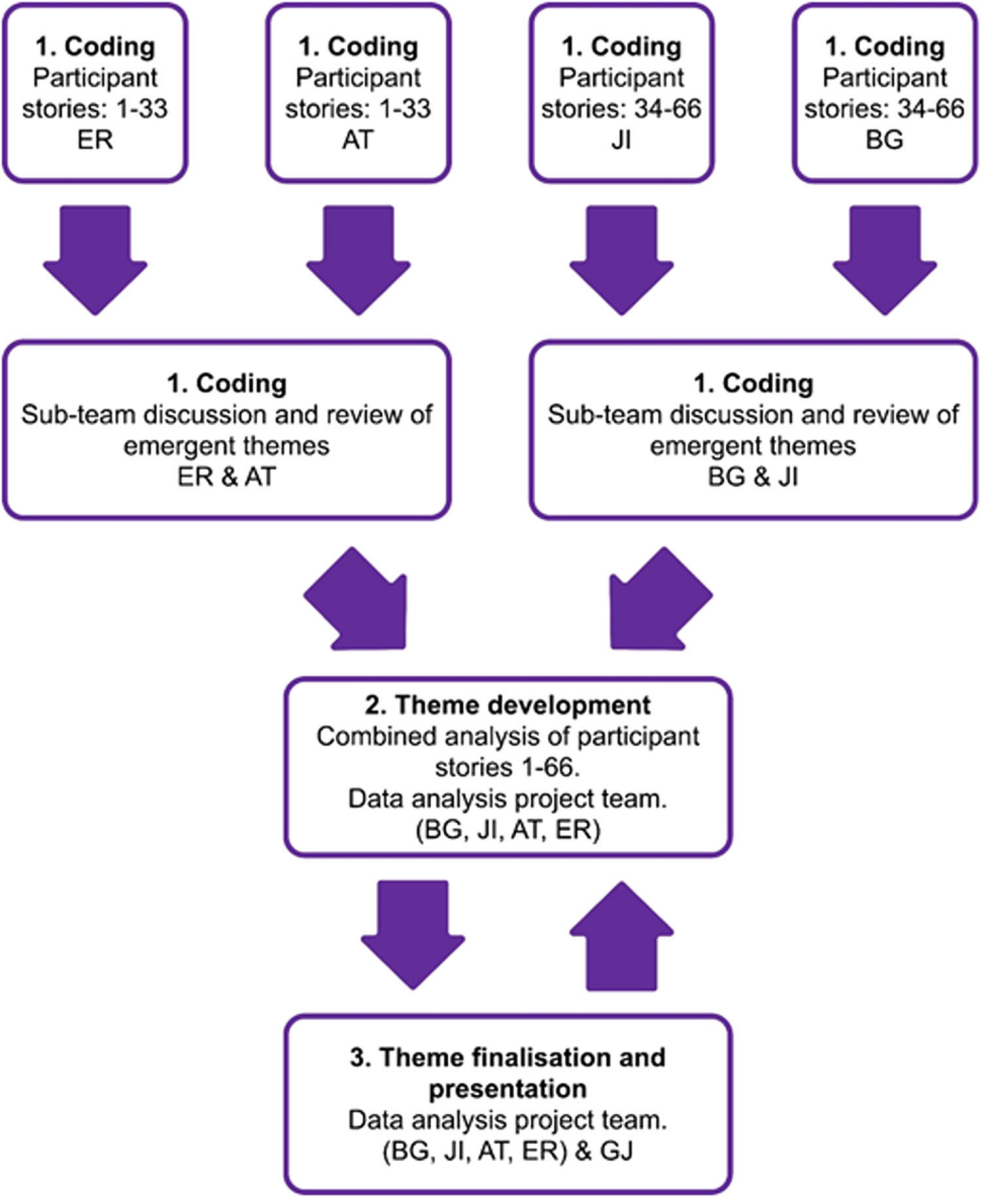
Process of data analysis.

Table 2 and Figure 2 present the method and the processes embedded into the analysis and the presentation of the themes. The method and processes establish and exhibit research trustworthiness,^23^ by providing objectivity, dependability and transferability.^18^

**Table 2:** Research process

	Format	Process
Part 1: Coding	The four project team members were divided into two subteams (E. R. + A. T. & B. G. + J. I.). Thirty‐three stories were allocated to each subteam for analysis.	**Individual**
		Each individual conducted a line‐by‐line review of their allocated 33 stories.
	Each subteam collaborated on a single Quirkos workspace.	Any relevant text was highlighted, and a ‘Quirk’ code was created on the workspace based on the content of the text.
		Texts perceived to be related to the same theme were added to existing Quirk categories.
		New Quirk codes were created when appropriate.
		Process was repeated a further three times to ensure data saturation of each story.^19^
		For emergent themes, codes were organized into loose clusters for discussions within each analysis subteam.
		**Analysis subpairs**
		Codes and emergent themes of the 33 stories were discussed in the collective subteams.
		Duplications were merged/removed, and a consensus of meaning was generated across each subteam.
		Showing objectivity and dependability.^18^
2. Theme development	Four project team members (B. G., J. I., E. R., A. T.).	**Project team**
	Collaboration was on a single Quirkos workspace.	The two workspaces were merged enabling the team to collectively discuss the codes generated by each subteam.
		A table of themes and a thematic map presented the key themes and subthemes enhancing the team's conceptual understanding of the data through visual organization^14^ and illustrated when data saturation had been reached with the development of no new themes.^19^
		The data codes and thematic map were collectively reviewed and discussed to establish coproduced themes.
		During coding, emergent Quirk categories were labelled with the language used by the participants. At this stage, the categories were relabelled where appropriate using inclusive language, to incorporate distinctions in the data.^12^
		A system of indexing was established, visually reviewing the categories to establish similarities, contrary and interconnectivity.
		The ‘drag and drop’ function of Quirkos was used to merge similar categories when duplicates were identified and designate categories as a ‘parent’ when subthemes emerged.
		Quotes assigned to each code were examined to ensure they were representative of the theme. Themes were removed or reassigned where appropriate.
		Discussion supported by the participant quotes ensured all decisions were based on a mutual agreement and reflective of the data.
		The table of themes and thematic map were updated to reflect the iterative changes arising from the group analysis.
		Showing objectivity and dependability.^18^
3. Theme finalization and representation	The four project team members (E. R., A. T., B. G., J. I.) plus G. J.	The development and agreement of the final themes were an iterative process occurring through a series of team meetings with all members of the project.
		The table of themes and thematic map were updated at each stage of discussion until the final themes and subthemes were agreed upon. All emergent themes and subthemes were supported by direct participant quotes.
		Showing transferability.^18^

## 3 RESULTS

**Figure 3:**
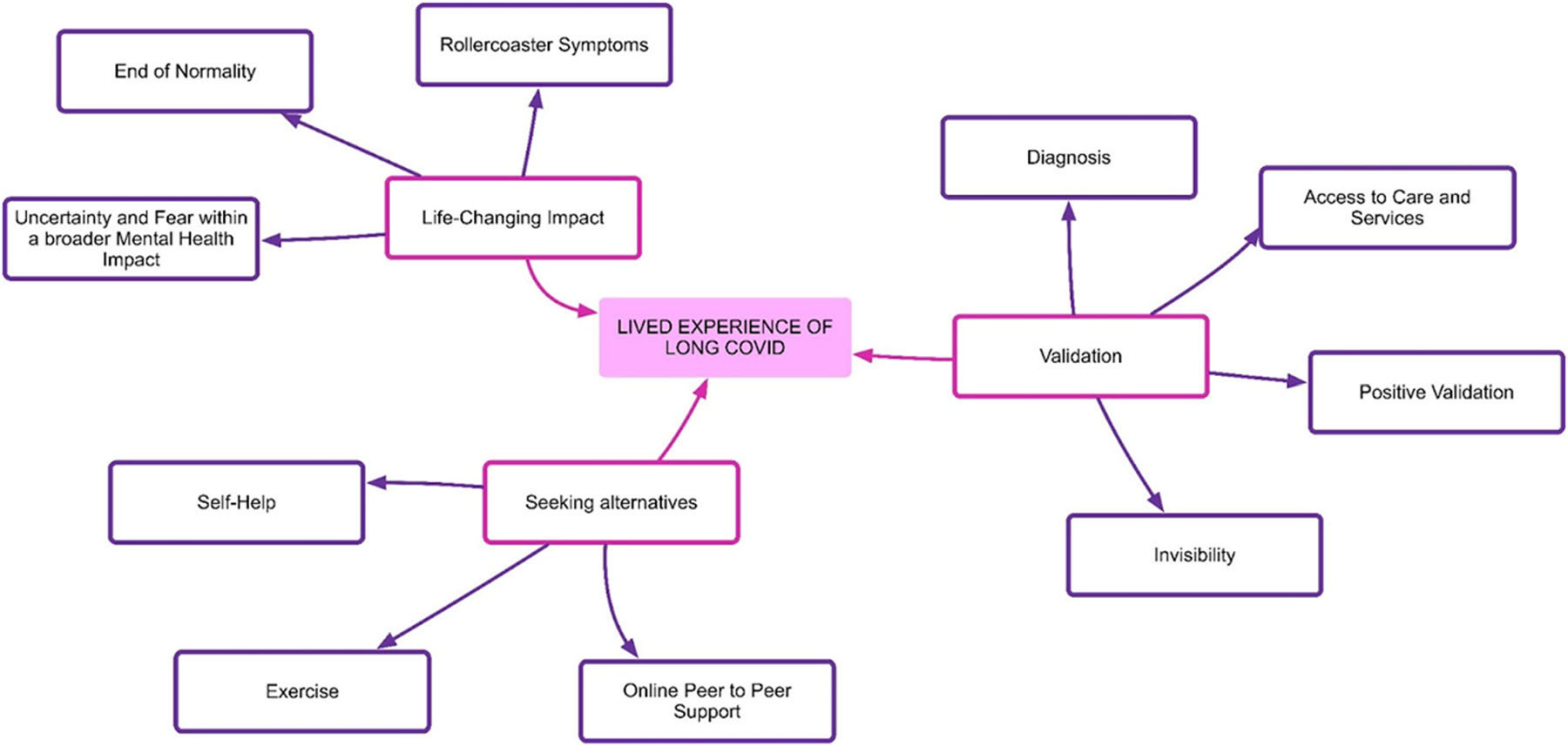
Key themes.

The overarching theme emerging from the analysis of the participants' stories depicts the challenges and complexities associated with the diagnosis and management of Long Covid before it was officially recognized. Three key themes emerged during the thematic analysis (1) Life changing (2) Validation and (3) Seeking alternatives (Figure 3).

### 3.1 Life changing

This theme reflects the sudden and significant physical impact of Long Covid on those with a confirmed or suspected diagnosis. This theme had three embedded subthemes: (i) end of normality, (ii) rollercoaster symptoms and (iii) uncertainty and fear within a broader mental health impact.

#### 3.1.1 End of normality

At the start of the pandemic, it was generally thought that a ‘mild’ Covid‐19 infection would only last 2 weeks. The narratives portray a different story, highlighting the severity of ‘milder’ nonhospitalized cases for young and healthy people, with previously fit and active lifestyles.

*Other than the mild asthma I was in great health before April 11th [2020]. I have run and completed 20 full marathons. I flew jets for a living. I'm not a wimp. But now I feel like I couldn't fight my way out of a wet paper bag*. Participant 31

*Before this, I was an extremely fit, active 31 year old who never had any health problems…. Everything is a struggle–eating, lifting cutlery, washing etc. My entire body is exhausted to the point that I have never functioned at all normally since the start, not even a basic normal… I want to get back to work and life and enjoyment. But I can't even look after myself, or function in even a normal way around my tiny flat*. Participant 7

As their symptoms and health issues continued, there came a realization that Covid‐19 could have a lasting impact on their health and lives.

*I tell people it is like living a half‐life. I can function but can't do what I used to. If I go out for a walk it means I have no energy for anything else*. Participant 59

*Everyone has a lot to lose with a change in their health, but I feel something like grief at the potential change in my physical health*. Participant 1

*I long for this to all go away*, [I] *am desperate to resume normal life again. Bringing me back to health that I knew before January, now appears a long and difficult road*. Participant 21

#### 3.1.2 Rollercoaster symptoms

The symptoms experienced by the participants were described as a ‘rollercoaster’, swinging between periods of good health and relapse.

*The rollercoaster of symptoms has been amazing in these 24 weeks, one week I would have fevers, the next week cough, the next week gastrointestinal symptoms, the other diarrhoea, the next brain fog, the next dry eyes, the other shortness of breath, the other heart palpitations and the list keep continues*. Participant 32

*This thing lets you know by knocking you right back down if you try too hard. It's as if it has a vindictive sort of intelligence that teases you into thinking that you're getting better*. Participant 42

*The uncertainty was exhausting. I had good days which made me hopeful and bad days which made me feel foolish for thinking I was in the clear*. Participant 28

#### 3.1.3 Uncertainty and fear within a broader mental health impact

Due to the unrelenting nature of ongoing Covid‐19 symptoms, the participants were fearful of the future and predicted a future of uncertainty and long‐term ill‐health, which would require further changes to their normal lives.

*I'm left with the prospect of a 4‐6‐10‐12 month covid‐recovery period due to impacts on my health whilst the covid was active*. Participant 40

*Truthfully, I was horrified. With the little bit of energy I had, I cried. I knew that from that point on, I nor anyone else had any idea what the next few days, weeks, months, or even years looked like for me*. Participant 28

There was evidence of the underlying mental health impact when people's lives had been so drastically affected by the physically devastating nature of a Covid‐19 infection.

*My life is completely upside down. I can't look back yet on this year since April when I caught COVID19 yet. I have to concentrate on my day when I get up and keep focusing on the future. The time will come when I can digest what's happened to me this year but I'm nowhere near ready to do that yet*. Participant 22

*This is now of course affecting my mental health and I feel low in mood at times, and struggling with living alone but also hate requiring other people's help. I am often too tired to communicate anyway. I hate being dependent*. Participant 7

### 3.2 Validation

This theme relates to how patients with confirmed or suspected Covid‐19 felt like they were not always heard or believed that their ongoing symptoms were related to a Covid‐19 infection. Within this theme are four subthemes where validation had an impact on their experience: (i) diagnosis, (ii) access to care and services, (iii) invisibility and (iv) positive validation.

When patients were not listened to, it created a plethora of missed learning and service delivery opportunities for the healthcare profession. It also had a significant impact on the care received by the participants. By not listening or hearing their concerns, the healthcare system left them vulnerable to both the physical and mental impact of the virus, the emotional impact of being left to cope alone and the psychological impact of not being taken seriously.

#### 3.2.1 Diagnosis

Symptoms that could lead to a diagnosis of Covid‐19 or Long Covid were not always taken seriously in part due to health services being overwhelmed, and, due to a lack of knowledge within the healthcare professions.

*The nurse, she told me I should stay home, and only if my lips turned blue, should I call for an ambulance. I felt all alone in my illness, as no health care provider I contacted seemed to regard what I was experiencing. No one ever said to me, ‘You might have Covid’, but rather they basically said, ‘… it must be something else’*. Participant 23

Many patients without a formal diagnosis persevered, without a doubt that their symptoms were related to Covid‐19.

*The past 7 months have been so devastating to both myself and my family. We have been alone with this–firstly when my symptoms lasted 6 weeks and no‐one believed that Covid symptoms could last that long and even more recently, when I've had to argue with GP's to refer me to specialists. Covid clinics are not readily accessible to sufferers and, certainly in my experience, long Covid is not something that all GP's are happy to acknowledge, especially without any positive test*. Participant 56

Later into the pandemic, as knowledge of the virus increased it was more likely that patients' symptoms would be recognized as Long Covid, but patients felt frustrated at not being able to retrospectively get any biomedical confirmation of having had a Covid infection.

*I had a test at 9 weeks and a test at 14 weeks, both negative but when I would have been positive, they weren't offering tests!*. Participant 9

*I will still never know for sure, as the antibody test I had done *>*6 months after the infection was negative, but the symptoms and ongoing signs of ‘long Covid’ leave with a high certainty that I had it*. Participant 50

For many of those participants who were not confirmed as a positive case, it meant their symptoms were never validated or accepted as an official case of Covid‐19. This was to have important consequences for the later stages of their illness trajectory and care delivery.

#### 3.2.2 Access to care and services

The participants were accepting that knowledge of the virus was still limited, but they were also frustrated at how repeatedly their ongoing symptoms were not taken seriously or taken as a symptom requiring a medical intervention that would lead to recovery.

*I told my Dr but they said they weren't aware of that being a Covid problem–every time I reported something they said they didn't know if it was related or not. I felt exasperated. *Participant 58

*It has felt like there was no understanding from the Doctors. I was having to send medical evidence, pictures etc. to them, for them to listen to me. My Doctors certificate says ‘numerous unexplained conditions’ how can this be?*. Participant 41

*My doctor didn't seem to know what to do with me, and said it sounded like panic attacks, eventually over the weeks he started to suggest Chronic fatigue…. there is still very little support*. Participant 19

#### 3.2.3 Invisibility

There was a subtheme of the impact of isolation and ‘invisibility’ when participants felt their symptoms were not believed, that in some ways their illness was not ‘real’ or could be altered by the behaviour of the participants themselves, rather than a condition with a medical cause.

*I am waiting on an antibody test and respiratory tests. Consultant said I am probably suffering from reconditioning through lockdown. This affected me badly. Nobody is listening. I feel debilitated and so depressed*. Participant 24

*I also feel angry, anxious and pissed off. Angry that people think covid is just mild for 2 weeks or death or ITU or just for older people. It is so, so wrong. I am angry when people give advice like maybe you aren't eating properly or you need to strengthen your legs. I couldn't be trying any harder, I am desperate to get back to life and work, but my body is not letting me*. Participant 7

*The ER doctor basically said I had had a panic attack. I had waited on that Covid ER ward for 5 hours, to have this doctor tell me people get over Covid in 2 weeks, my symptoms were vague, and I must be stressed and depressed because of the pandemic*. Participant 23

#### 3.2.4 Positive validation

On the other hand, though far less prominent in our analysis, were patients whose health‐illness experiences were validated by HCPs, often generating a sense of reassurance and relief. Participants felt happier when doctors shared uncertainty rather than were dismissive.

*Throughout my rollercoaster journey, my GP has been understanding. I have had more contact with my surgery in the last 4 months than I have had in my life. I keep regular check ins when things worsen. I have had an inhaler, a course of antibiotics, bloods and a chest X‐ray. Medical experts are learning as they go about the impact of the virus and at the moment there is no treatment/magic wand*. Participant 25

*After a week thinking things would improve I rang the GP I finally got a face to face appointment. And a very thorough exam. I felt listened to and felt happier*. Participant 29

### 3.3 Seeking alternatives

The final theme reflects participants' desires to seek alternatives to formal healthcare during their illness and includes the subthemes: (i) online support groups, (ii) self‐help and (iii) exercise.

#### 3.3.1 Online peer to peer support

Long Covid emerged as a very enigmatic and complex illness at a very difficult time for overwhelmed health services. A significant online community for Long Covid support arose during the pandemic, developing at a speed that was impossible for formal healthcare support to catch up with, especially given the restrictions on physical movement and social distancing. These online communities appeared to provide invaluable sources of validation and support.

*It's a relief to me that everything I've gone through in the last eight months is finally being validated by other people now having very similar experiences as mine post‐covid. I've felt very alone in this whole experience to this point*. Participant 20

*So ultimately, I have had to struggle through alone, figuring out what works and what doesn't. It's been the kindness of people through professional networks or online peer support that has helped, more than structured services for people living with such disability*. Participant 55

The internet was a powerful player in supporting patients who may have otherwise felt let down by formal health care.

*Why was it the doctors and hospital were still not aware of these when I was able to find out the information on‐line? Why would no one see me but only talk over the phone, why was I sending them the information and links from the internet before they listened to me!?*. Participant 21

*The thing seemed endless. My GP was good at treating symptoms and was the first person to tell me that ‘covid insomnia' was ‘a thing’, but really had no idea what was going on with me. I had no idea that other people were going through the same thing until the mainstream media started to highlight long‐haul covid‐19 and linked to some Facebook groups which were a lifesaver*. Participant 13

#### 3.3.2 Self‐help

It was perhaps not surprising that patients were willing to try diets, medications and supplements in a desperate bid for self‐improvement when diagnostic testing and treatment for Long Covid were unavailable, even a clinical diagnosis and professional validation were hard to come by.

*My GP tried to be supportive but has refused a referral to a ‘long Covid clinic’ as they say there is not one locally. I am taking multivitamins, folic acid, B‐12, cod liver oil, Vit D, CoQ10 and turmeric religiously in an attempt to try and help myself*. Participant 61

*Every day I have tried to be positive and think I can outsmart or positive think my way out of this, however despite doing everything I can regarding activity, emotional support from friends, graded exposure to functional life, vitamins and supplements, giving up caffeine, healthy diet choices, however these symptoms persist*. Participant 17

#### 3.3.3 Exercise

Exercise emerged as a complex subtheme presenting as an unknown factor in recovery, given that in other postviral conditions exercise or pushing through could make things worse. For most, it seems incorporating exercise into Covid‐19 recovery is a delicate balancing act.

*I'm still struggling to have the confidence to push myself to make gains with my physical fitness, as I still don't fully understand the balance between what might be a trigger–as some days I can get away with more… I'm terrified of an avalanche of chronic fatigue, like I'm standing on the edge of a cliff with no control over when and if I will fall*. Participant 1

*I have had to abstain from caffeine, alcohol and my usual exercise (running and cycling) for the duration of my illness so far. As well as getting back to full time work I have been trying to improve my fitness again with short walks, limited by ongoing symptoms*. Participant 60

## 4 DISCUSSION

Long Covid is a novel and enigmatic illness.^8^ It has been a well‐documented struggle for HCPs and patients alike to achieve recognition of Long Covid by national agencies and governments.^4^ Our analysis of patient stories from the beginning of the Covid‐19 pandemic illustrates the lived experience of this struggle for recognition. Patients with Long Covid were struck by its life‐changing impact on their health yet were not always listened to or believed, leading them to yearn for validation as a vital stepping stone to engaging in the management of their illness. While Long Covid remained undefined and unrecognized, patients in their invisibility were compelled to make sense of their experience largely through online networks and were prepared to try informal, unregulated treatments and lifestyle shifts in an attempt to improve their health.

### 4.1 Comparison with existing literature

This study is part of a small but important group of studies that have documented the lived experience of Long Covid in the UK.^2^, ^10^, ^24^, ^25^, ^26^, ^27^ As we are not the first group to publish our results, we have the benefit of being able to place our findings in the context of previous literature to build an overall picture from multiple perspectives. Findings from this, explicitly community‐sourced data, show very similar findings to those from researcher‐led studies, adding to the validity of the body of evidence.

Overall, this body of evidence highlights the two areas in which Long Covid is particularly challenging to live with. Firstly, is that regardless of the severity of an initial Covid infection, recovery is not always straightforward as government advice initially outlined, and instead for those who develop Long Covid it is a serious, uncertain and confusing illness with a life‐changing physical impact. The experience of the illness is chaotic, episodic and ongoing, and individuals are prevented from returning to their previous healthy selves, causing a significant biographical disruption.^2^, ^24^, ^26^

Secondly, Long Covid has characteristics of a hidden or invisible illness, which causes stigma, shame and epistemic injustice. Long Covid is ‘hard, heavy work’^24^ not only in managing life‐changing symptoms but also in having to interact with a healthcare system that does not routinely believe your testimony, or recognize your illness experience. The epistemic injustice is like hot lava running through all the Covid‐19 lived experience research, where, ‘clinician[s] did not recognise their condition, did not believe that it existed, did not know how to diagnose it, did not empathise or acknowledge their suffering, [and] did not know how to manage it’.^2^

Invisibility and epistemic injustice are not unique to any one illness yet can be linked with other diseases shown to have low ‘disease prestige’,^28^ which are characterized by not being organ‐specific, do not have objective diagnostic signs and efficient therapeutic options are not always available.^12^ Other invisible illnesses include fibromyalgia, endometriosis, depression, chronic pain and depression yet the most commonly associated with Long Covid is the similarity in experience to ME/chronic fatigue syndrome (CFS).^2^, ^24^, ^27^ Qualitative research into the lived experience of ME/CFS has highlighted how dominant illness models are a barrier to the HCP–patient relationship.^29^ As Bayliss et al. (2014, p. 7)^29^ concludes, ‘the biomedical approach, which is central to the medical curriculum, leads many health care professionals to conclude that there is no real illness [in ME/CFS] as there is currently no identifiable pathology’.

The ways in which both clinicians and patients respond and/or try to mitigate this uncertainty can impact the way appropriate care and support are delivered and managed.^30^ For example, if a patient presents with debilitating fatigue and brain fog post suspected Covid, there can be a contest between different models explaining the disease process at work (organic vs. psychosocial). The patient's testimony is vital to the consultation as there is no available epistemological authority from a biomedical explanation or test, yet clinicians can somatize these symptoms as seen in our analysis (blame deconditioning or stress) and downgrade the patient's credibility. This is epistemic injustice as described in the literature^12^, ^31^ and can lead to a climate of distrust as seen in the lived experience of Covid‐19.

Covid's paradox created the perfect storm for patients and HCPs to struggle to navigate Long Covid. HCPs are increasingly vulnerable to frustration, stress and burnout. During the pandemic, HCPs have been working in the context of ‘moral injury’ at having to care for patients with a ‘near incurable virus’, placing loved ones at risk, extended shifts and workplace chaos—and they also face an evidence gap in supporting patients with Long Covid.^26^ Acknowledging the impact of this evidence gap, the research speaks of the fundamental importance of bearing witness and active listening, even in the face of medical uncertainty.^2^ Several Long Covid studies have included HCPs who have found living with Long Covid transformative, in that they are now ‘better able to care for and empathize with patients with ongoing and unexplained symptoms’.^27^ Researchers in ME/CFS show that HCPs feel that despite changing attitudes, their medical education failed to equip them with the knowledge and therapeutic skills required to diagnose and manage CFS/ME according to current guidelines.^29^ Addressing this gap in medical education must be a priority as guidelines for complex, chronic illnesses like ME and Long Covid require a compassionate and empathic patient‐centred approach fundamental to which are advanced communication and interpersonal skills.^15^, ^32^

### 4.2 Implications for research and practice

Both within this analysis and earlier qualitative studies on Long Covid there is evidence of HCPs successfully diagnosing and managing Long Covid and building a meaningful therapeutic relationship.^2^, ^10^, ^24^, ^27^ Evidence from the CFS/ME literature also points to these successes as ways of showing how barriers can be overcome when HCPs take, ‘a more flexible, biopsychosocial approach, building a positive, collaborative therapeutic relationship with their patient’.^29^ As patient representatives, we would argue for more epistemic humility from healthcare providers, as an approach that calls for partnership and dialogue to underpin the trust that is essential for a therapeutic relationship.^31^ Beyond the scope of this paper, we would also argue for significantly advancing our ability to articulate the marginalization of patients as knowers, through more discussion and reflection of epistemic injustice in research, education and clinical practice. This discussion must also reflect and explore already existing inequalities in society to ensure they are not further exacerbated in dealing with an invisible illness.

### 4.3 Strengths and limitations

The Covid Recovery Collective was set up by two of the authors of this study (J. I. and E. R.) who may demonstrate bias in the analysis as they could hope to validate their initiative through the research. Any bias caused by the developers' involvement is mitigated through the process of analysis employed and the composition of the research team, which included a patient who has experienced Long Covid and myalgic encephalopathy/encephalomyelitis (ME) independent of the website, and an independent researcher and academic supervisor.

A strength of our study was the unique data set, where participants voluntarily submitted their narratives as they were living through Long Covid before it was officially recognized. This method of capturing lived experience qualitative data is implicitly unbiased by any research process, which may have a retrospective element, or a particular research focus or agenda, and/or has actively set out to identify and recruit predefined samples of patients. A limitation however is that a diagnosis of Covid‐19 and/or Long Covid are not always clinically proven, either due to a lack of testing or diagnostic makers, and at this very early stage of the pandemic we have relied on the patients who have self‐reported their diagnosis. Some patients may not have had Covid‐19 but another illness. As this study did not start as a research project, the sample is not selected to be representative of those experiencing Long Covid but does include a mix of age, gender and ethnicity (see Figures A1, A2, A3, A4). The sample is naturally biased towards those with access to the internet, and those seeking validation and support online, which may not be representative of the whole Long Covid population.

## 5 CONCLUSION

Covid‐19 caused an unparalleled global impact as leaders worldwide continue to work to reduce infection and death rates from this novel respiratory virus. Behind the front lines, there has been a well‐documented battle to gain recognition of the more long‐term complications of Covid‐19, which can cause significant disability and chronic ill health, known as ‘Long Covid’. What we can learn from individuals who have lived with Long Covid is the sudden and life‐changing impact of the condition, which, given its novel and enigmatic nature, did not fit within existing biomedical understandings of a Covid‐19 illness. This meant that many patients struggled to be heard and yearned for validation of their illness, which left them feeling invisible and lacking the services and support they needed to manage the illness. Without objective indicators of the disease process to fall back on, Long Covid patients rely on having their illness testimonies believed and taken seriously to access treatment and support in a therapeutic relationship based on trust. Long Covid patients have been systematically denied epistemic justice in the testimonies of their illness, which has increased the suffering caused by their illness. As patient representatives in citizen‐based research, we have outlined the importance of epistemic humility in dealing with Long Covid. Looking forward, and in line with current guidelines, Long Covid patients' access to services and support can be improved when, at all levels of society, we acknowledge and validate the lived experience of people with invisible illnesses. It is vital to support and empower patients and health care providers alike to build positive and collaborative therapeutic relationships to navigate care.

## AUTHOR CONTRIBUTIONS

**Jane Ireson**: Conceptualization, methodology, formal analysis, writing–original draft, review and editing, funding acquisition, project administration. **Amy Taylor**: Methodology, formal analysis, writing–original draft, writing–review and editing. **Ed Richardson**: Conceptualization, software, formal analysis, writing–review and editing, project administration, data curation. **Beatrice Greenfield**: Formal analysis, writing–review and editing. **Georgina Jones**: Conceptualization, methodology, formal analysis, writing–review and editing, supervision.

## CONFLICTS OF INTEREST

Jane Ireson and Ed Richardson are the co‐creators and co‐owners of the Covid Recovery Collective (noncommercial) https://covid19-recovery.org/. Other authors declare no conflicts of interest.

## Data Availability

The data that support the findings of this study are openly available at https://covid19-recovery.org/.

## 1

See Figures A1, A2, A3, A4.
